# An Intergenerational Service-Learning Model for Younger and Older Learners: Linking Community and Classroom

**DOI:** 10.1093/geroni/igaa057.1819

**Published:** 2020-12-16

**Authors:** Tamar Shovali

**Affiliations:** Eckerd College, St Petersburg, Florida, United States

## Abstract

The intergenerational reflective service-learning program titled Mentor Up is intentionally integrated within gerontology education. Mentor Up, a series of one-on-one technology training workshops, connects younger with older learners in the community while building on course content. Groups of students are paired with the Academy of Senior Professionals at Eckerd College (ASPEC) members, a continuing education organization whose 300+ members are committed to lifelong learning. Members are fully integrated into the course, serving as mentors for students. These class sessions support students in planning for Mentor Up service at a local CCRC and promote positive contact between students and retired community members. ASPEC members also participate with students in a significant reflective component concluding the course – in which students and older learners work together to write a script, record, and present a video essay addressing aging stereotypes. Considerations for planning, classroom integration, community connection, and project reflections will be discussed.

